# Prevalence of Hepatic Encephalopathy from a Commercial Medical Claims Database in the United States

**DOI:** 10.1155/2021/8542179

**Published:** 2021-06-08

**Authors:** Aniruddha Potnis, Susan VanMeter, Jan Stange

**Affiliations:** Mallinckrodt Pharmaceuticals, Hampton, NJ, USA

## Abstract

**Introduction:**

Hepatic encephalopathy (HE), a complication of cirrhosis, is associated with increased healthcare resource utilization and mortality, and impaired quality of life. Information on the prevalence of HE in the US general population is limited.

**Methods:**

Prevalence of HE was estimated by sequential stepwise data analysis of the Symphony Health anonymized patient-level data (APLD) claims database. First, patients ≥ 18 years with International Classification of Diseases ninth/tenth edition, clinical modification (ICD-9/10-CM), and codes for cirrhosis from 2018 medical and hospital claims were used to estimate prevalence of cirrhosis within the data set and number of patients with cirrhosis in the US general population. Second, patients diagnosed with cirrhosis in the APLD data set from 2015–2016 with an HE ICD-9/10-CM code within 1 year of cirrhosis diagnosis were used to deduce the prevalence of HE within the data set and estimate the number of patients with HE in the US general population. Last, US DiagnosticSource data on serum ammonia level laboratory results measured within ±2 days of a confirmed HE event were merged with the APLD HE data set, then applied to the US general population.

**Results:**

Medical and hospital claims data were available for 272,256 patients with cirrhosis in 2018. An estimated 536,856 US adults had a diagnosis of cirrhosis (prevalence of 0.21%) in 2018. This proportion applied to the estimated number of patients with cirrhosis in the United States resulted in a prevalence estimate of 201,858 cirrhosis patients with HE in 2018. When factoring in serum ammonia data, prevalence was conservatively estimated as approximately 196,000 cirrhosis patients with HE and serum ammonia levels > 21 *μ*mol/L.

**Conclusions:**

In this longitudinal cohort–based study, it was estimated that ≈202,000 patients had HE in the United States in 2018, representing a considerable burden to society and payers.

## 1. Introduction

Hepatic encephalopathy (HE) is a complication of cirrhosis that is associated with increases in healthcare resource utilization and patient mortality and a decrease in quality of life [1–3]. In more severe HE, rehospitalization is common, although management of these patients can reduce readmission rates [4, 5]. Despite appropriate therapy, research suggests episodes of HE may be associated with cumulative cognitive deficits [6]. Reducing the risk of recurrence of HE events and the resulting burden of disease would be valuable to payers and the healthcare system overall. To better characterize the burden of HE, an estimate of the disease prevalence is critical. However, to date, prevalence estimates for HE in the US general population have not been reported.

Hepatic encephalopathy pathogenesis is not completely understood but involves ammonia as a key neurotoxic substance. Due to liver dysfunction, serum ammonia levels increase and cross the blood–brain barrier; the ammonia within the brain stimulates the release of inflammatory signals from astrocytes, which swell and produce cerebral edema [7, 8]. Currently, no HE prevalence estimates present data with and without associated serum ammonia concentrations within the literature. Because elevated ammonia levels are a key contributor to HE and a potential therapeutic target, it could be informative to understand the distribution of serum ammonia levels in cirrhosis patients with HE.

Herein, the authors report US prevalence estimates for HE in adult cirrhosis patients based on analysis of an anonymized, longitudinal, patient-level claims data set. A further analysis combines the prevalence results with serum ammonia laboratory data.

## 2. Methods

### 2.1. Data Source and Study Design

Anonymized patient-level data (APLD) from Symphony Health claims were used in this retrospective and cross-sectional, cohort-based analysis of the 1-year–period prevalence (hereafter referred to as prevalence). The Symphony Health claims database represents approximately 284 million patient lives from January 1, 2014, to December 31, 2018. This data set includes anonymized patient identifiers, hospital stays with associated diagnoses and procedures, outpatient visits with associated diagnoses and procedures, and pharmacy dispensing activity. Furthermore, this data set includes 1500 claims from the Centers for Medicare and Medicaid Services for medical office data, UB-04 claim forms for hospital data, National Council for Prescription Drug Programs claims for prescription data, and pharmacy data from specialty pharmacy shipment information. In addition, deidentified patient diagnostic laboratory data from DiagnosticSource (derived from Symphony Health IDV®) were used to match HE events to serum ammonia level laboratory data. Anonymized patient-level data claims are Health Insurance Portability and Accountability Act compliant.

### 2.2. Subjects

The cirrhosis prevalence analysis included adult patients (≥18 years of age) within the APLD database with medical-claim or hospital-claim activity from at least one quarter of 2018 and an International Classification of Disease (ICD), ninth and tenth revisions, clinical modification (ICD-9-CM or ICD-10-CM) diagnosis code for cirrhosis (Table 1). Patients without a recorded birth year, or with a diagnosis code of 571.6 (biliary cirrhosis, as previously described [9]), were excluded from the prevalence analysis. During the 12-month observation period (January 1, 2018, to December 31, 2018), an activity check was conducted: included patients were required to have had a medical, hospital, or prescription claim transaction involving either a diagnosis, prescription activity, or surgery/procedure code to ensure survival.

For the HE prevalence analysis, adult patients (≥18 years of age) within the APLD database were identified if they received a first cirrhosis diagnosis from January 1, 2015, to December 31, 2016. The index date was defined as the earliest date of cirrhosis diagnosis within the observation period. Any additional diagnoses for the same patient were not considered for this analysis. Patients were required to have at least 12 months of claims preindex date; the purpose of this lookback period was to ensure they did not have a prior diagnosis of HE and in fact were newly diagnosed. Patients were excluded from the analysis if they had a diagnosis code for HE prior to the index date or had claims activity in fewer than 2 quarters of the observation period. In the resulting cirrhosis analysis cohort, the number of prevalent cases of HE ICD-9-CM and ICD-10-CM codes (Table 1) within 1 year of cirrhosis diagnosis (index date) was analyzed during the observation period.

Real-world, patient-level, diagnostic laboratory data were used to assess the distribution of ammonia levels within identified patients with HE. Serum ammonia values were obtained from DiagnosticSource for the subset of patients with an HE diagnosis claim used in the prevalence analysis. Laboratory data were merged into the APLD data using unique patient identifiers to describe patients (≥18 years) with serum ammonia levels measured during or within ±2 days of a recorded HE event.

To ensure laboratory data were reflective of the disease severity for the larger HE patient population, the number of HE events was compared between the laboratory data set and the APLD data. The HE cohort in the APLD database was defined as all patients with first HE diagnosis (index date: 2016), and the number of events was defined by examining a longitudinal duration beginning 2 years prior to the index date and ending 2 years following the index date. The laboratory cohort included patients with HE that had documentation of serum ammonia level measured within ±2 days of an HE event. The rate of HE events from April 1, 2017, to March 1, 2019, was determined for the laboratory cohort.

### 2.3. Statistical Analysis

Counts of patients were aggregated by medical or hospital claim into age groups (18–25, 26–34, 35–44, 45–64, and ≥65 years) in the cirrhosis prevalence analysis (2018). Prevalence was calculated by dividing the number of patients within each age group with a diagnosis code by the total number of individuals within each group. The age-specific prevalence proportions were then multiplied by the US population by age in 2018 as reported by the US Census Bureau. A weighted sum of US patients with cirrhosis was calculated from the hospital and medical claims prevalence estimates.

**Figure 1:**
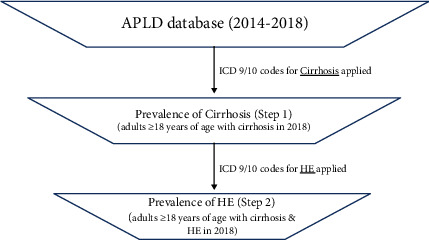
Estimation of HE prevalence in patients with cirrhosis. APLD: anonymized patient-level database; HE: hepatic encephalopathy; ICD: International Classification of Diseases.

The prevalence of HE among patients with cirrhosis was calculated by dividing the number of patients with an HE diagnosis code by the number of patients with a first cirrhosis diagnosis code in 2015–2016. The resulting HE prevalence was then applied to the estimated number of US cirrhosis patients to define the subpopulation of patients with HE (2018).  Figure 1 depicts the step-wise analysis for HE prevalence.

To analyze the distribution of ammonia levels in patients with HE, the proportion of patients with HE was reported for prespecified serum ammonia level groups. The prevalence of patients was then applied to the 2018 estimate of cirrhosis patients with HE to determine the number of patients within each prespecified serum ammonia level group.

## 3. Results

### 3.1. Cirrhosis and Hepatic Encephalopathy Prevalence

Overall, 272,256 patients with cirrhosis claims were identified from the APLD Symphony Health database in 2018 (Table 2). Cirrhosis patients in the analysis were most likely to be aged 45 to 64 years (53%), male (54%), and have Medicare coverage (52%; Table 2). The prevalence of cirrhosis was highest in individuals 45 to 64 years of age (0.56% and 0.29% based on hospital and medical claims, respectively; Table 3). When applied to the US population of patients with health insurance in 2018, 792,184 and 432,991 patients were estimated to have cirrhosis based on hospital and medical claims, respectively. The APLD claims database comprised 17.97% of all hospital claims and 44.18% of all medical claims. Accounting for the source data, the weighted sum of the number of patients with cirrhosis in the United States in 2018 was 536,856, resulting in a prevalence of 0.21%.

Within the APLD database, 99,004 patients were first diagnosed with cirrhosis from 2015–2016. It was determined that 37.6% (*n* = 37,214) of those patients with cirrhosis received a diagnosis of HE within 1 year of their respective index date of cirrhosis diagnosis. This proportion applied to the estimated number of patients with cirrhosis in the United States resulted in a prevalence estimate of 201,858 cirrhosis patients with HE in 2018.

### 3.2. Serum Ammonia Analysis

**Figure 2:**
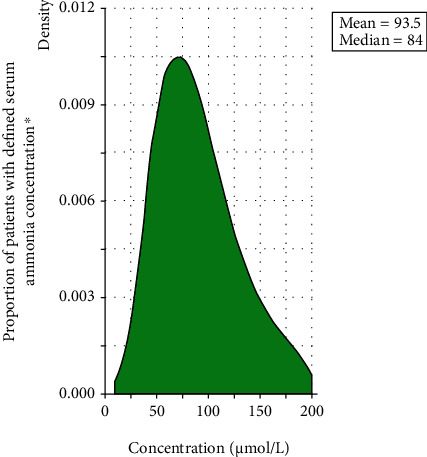
Serum ammonia distribution in cirrhotic patients with HE measured within ±2 days of a confirmed HE event. HE: hepatic encephalopathy. ^∗^*N* = 11,113 patients.

Within 11,113 patients with HE who were identified as having serum ammonia data (the other 26,101 patients did not have ammonia data), serum ammonia levels exhibited a mean concentration of 93.5 *μ*mol/L (Figure 2).  Table 4 highlights the proportion of patients above or below the serum ammonia level groups. Fewer than 200,000 cirrhosis patients with HE were estimated to have a serum ammonia level greater than 21 *μ*mol/L (*n* = 196,191).

Comparison of patients with HE in the Symphony Health APLD claims database and the serum ammonia data set demonstrated that the distribution of patients by number of HE events was similar (Table 5), suggesting that the 2 populations had similar clinical characteristics. The majority of patients in both cohorts experienced more than 1 HE event, with the largest proportion experiencing 5 or more events (34.2%, *n* = 20,225 APLD claims cohort and 31.8%, *n* = 1970 serum ammonia analysis cohort).

## 4. Discussion

This analysis offers a unique step-wise approach to HE prevalence estimation for the general public in the United States. The process began with the prevalence estimate of liver cirrhosis in adult patients (0.21%) in 2018 from the Symphony Health APLD database, which was similar to previously reported cirrhosis prevalence estimates in the literature (range 0.24%–0.27%) that used employer-based health insurance claims and data from the National Health and Nutrition Examination Survey [9, 10]. Previous studies on liver cirrhosis prevalence did not evaluate the prevalence of HE in the US general population. This analysis revealed a 37.6% prevalence of HE in patients with cirrhosis, which translates to an estimated 201,858 patients with cirrhosis and HE in 2018. The prevalence analysis included both medical and hospital claims data, reflecting both outpatient and inpatient data that should capture the entire range of disease severity of HE anchored from the first cirrhosis diagnosis. Overall, we estimated the prevalence of HE to be approximately 202,000 people within the United States. When merged with laboratory data, it was estimated that approximately 196,000 patients with HE had a serum ammonia level > 21 *μ*mol/L.

To the knowledge of the authors, no publication has considered the prevalence of HE relative to an association with ammonia levels. The use of serum ammonia levels in our analysis may be seen as controversial. While we acknowledge there is evidence within the literature that serum ammonia levels are not always predictive of outcomes and severity of HE at lower levels [11], there has still been support of the correlation between ammonia levels and the severity of the disease [12]. Ammonia levels have been shown to contribute to the risk of severe HE [13]; however, it is known that the etiology of HE comes not only from inflammation and increased ammonia levels due to cirrhosis but also from other metabolic disturbances resulting from liver disease, including other neurotoxins that may lead to an HE diagnosis [14, 15].

We used a serum ammonia level of >21 *μ*mol/L as a cutoff for an abnormal concentration suggestive of HE. However, there is no universally accepted serum ammonia concentration that is considered to indicate HE. In 1 study, Ong and colleagues noted that even among their patients who were without clinical signs or symptoms of HE (encephalopathy grade 0), 69% (20 of 29 patients) had total ammonia levels greater than the local upper limit of normal (47 *μ*mol/L) [16]. In another study, Gundling and colleagues determined the sensitivity and specificity of venous ammonia levels to diagnose HE by using the West Haven criteria and the critical flicker frequency test (a psychophysiologic test) [17]. Normal values were defined as ammonia levels of 12 to 55 *μ*mol/L. Hepatic encephalopathy was assumed when ammonia levels were greater than the normal limit of 55 *μ*mol/L; using that threshold, the diagnostic accuracy and 95% confidence intervals (CIs) of serum ammonia were determined to be 59.3% (45.7%–71.9%), sensitivity was 47.2% (30.4%–64.5%), and specificity was 78.3% (56.3%–92.5%). The positive predictive and negative predictive values (95% CI) were 77.3% (54.6%–92.2%) and 48.6% (31.9%–65.6%), respectively. Thus, while elevations of serum ammonia may increase the probability that HE is present, low ammonia concentrations are insufficient to eliminate the possibility of HE [17]. Nonetheless, the presence of ammonia levels of >21 *μ*mol/L in approximately 97% (196,191/201,858) of our sample of patients with cirrhosis and HE seems consistent with a role for ammonia in the etiology of HE.

Limitations of this study include the use of 2 different data sets to analyze serum ammonia levels and HE events. These 2 data sources represent different populations that could have had different characteristics. Logistically, it was not possible to obtain older laboratory data. To minimize this limitation, the distribution of HE events in both data sets was analyzed to assess disease severity and found them to be comparable, albeit not identical. Additionally, the prevalence identified in this analysis might not exactly match the overall population due to disparities in insurance coverage. Analysis of the data revealed that not all patients with HE events had ammonia levels measured, which may have been due to a variety of reasons (e.g., socioeconomic status and type of insurance); the timely and accurate measurement of ammonia also can be challenging and have variable reliability [2]. Furthermore, uninsured patients were not available for analysis in our data set of insurance claims. Finally, data were obtained from administrative claims, and diagnosis was determined from an ICD-9-CM or ICD-10-CM code submission; thus, it is not possible to distinguish between overt and covert forms of HE. Although the data set does not allow us to distinguish between these 2 forms, both outpatient and inpatient diagnosis codes were recorded, suggesting that a spectrum from less severe HE (i.e., minimal HE) to more severe HE (i.e., overt HE) was observed. Tapper et al. have recently described a validated ICD-10-CM algorithm for claims analysis to identify HE from administrative data [18]. This approach involves identifying HE in patients with cirrhosis and based on recorded use of either lactulose or rifaximin. This algorithm was unavailable at the time of our analysis, but might have provided an additional means of confirming the presence of HE in our population of patients with cirrhosis.

Overall, this analysis confirmed previous literature estimates for the prevalence of patients with cirrhosis. Subsequently, the authors identified a large proportion of cirrhosis patients with HE within 1 year of cirrhosis diagnosis. The total estimated prevalence of HE in patients with cirrhosis in the United States in 2018 was approximately 202,000.

## Figures and Tables

**Table 1:** Identifying ICD-9-CM and ICD-10-CM Codes.

Diagnosis code	Description	ICD version number
Cirrhosis		
K74.60	Unspecified cirrhosis of liver	10
K70.31	Alcoholic cirrhosis of liver with ascites	10
571.2	Alcoholic cirrhosis of liver	9
K70.30	Alcoholic cirrhosis of liver without ascites	10
K74.69	Other cirrhosis of liver	10
K71.7	Toxic liver disease with fibrosis and cirrhosis of liver	10
K74.5	Biliary cirrhosis, unspecified	10
571.5	Cirrhosis of liver without mention of alcohol	9
K74.4	Secondary biliary cirrhosis	10
571	Chronic liver disease and cirrhosis	9
K74.3	Primary biliary cirrhosis	10
Hepatic encephalopathy		
K72.11	Chronic hepatic failure with coma	10
K72.01	Acute and subacute hepatic failure with coma	10
K72.10	Chronic hepatic failure without coma	10
K72.00	Acute and subacute hepatic failure without coma	10
K72.91	Hepatic failure, unspecified with coma	10
572.2	Hepatic encephalopathy	9
K70.41	Alcoholic hepatic failure with coma	10
K72.90	Hepatic failure, unspecified without coma	10
K70.40	Alcoholic hepatic failure without coma	10

ICD: International Classification of Diseases.

**Table 2:** Characteristics of patients with cirrhosis identified in the APLD claims database.

Parameter	Number of patients
Total number of patient claims in the United States	272,256
Medical claims	234,780
Hospital claims	190,697
Age (years) (*n*)	
18–25	991
26–34	5106
35–44	16,824
45–64	143,218
≥65	106,117
Sex (*n* (%))	
Female	125,340 (46)
Male	146,907 (54)
Blanks	9 (0)
Primary payer (*n* (%))	
Medicare	142,443 (52)
Medicaid	62,159 (23)
Government	4120 (2)
Commercial	56,067 (21)
Cash	2468 (1)
Others	2884 (1)
Blanks	2115 (1)

APLD: anonymized patient-level data.

**Table 3:** Estimation of cirrhosis prevalence rates by age group and projection to estimated cirrhosis prevalence in the US population.

Age group (years)	Estimated number of patients with cirrhosis from APLD database		Total number of patients in APLD database		Estimated prevalence from APLD database (%)		Total US population (2018)∗	2018 US cirrhosis prevalence estimates	
	Hospital claims	Medical claims	Hospital claims	Medical claims	Hospital claims	Medical claims		Hospital claims	Medical claims
18–25	675	867	4,821,638	12,847,208	0.01	0.01	35,063,183	4909	2366
26–34	3582	4486	5,961,467	15,458,294	0.06	0.03	41,091,493	24,690	11,925
35–44	11,394	14,804	6,423,299	17,317,948	0.18	0.09	41,277,888	73,221	35,286
45–64	96,276	128,013	17,345,958	43,817,387	0.56	0.29	83,904,335	465,698	245,127
≥65	70,645	95,499	16,560,408	36,208,191	0.43	0.26	52,431,193	223,666	138,287

APLD: anonymized patient-level data. ^∗^US population stratified by age group available at https://www.worldometers.info/demographics/us-demographics/.

**Table 4:** Serum ammonia levels in patients with HE.

Serum ammonia level range (*μ*mol/L)	Number of patients (%) with serum ammonia		Cumulative estimated number of patients with serum ammonia > corresponding level range in 2018^∗^
	≤ Corresponding level range	> Corresponding level range	
0–10	6 (0.05)	11,107 (99.9)	201,749
11–20	48 (0.43)	11,059 (99.5)	200,877
21–30	258 (2.32)	10,801 (97.2)	196,191
31–40	563 (5.07)	10,238 (92.1)	185,964
41–50	879 (7.91)	9359 (84.2)	169,998
51–60	1088 (9.79)	8271 (74.4)	150,235
61–70	1127 (10.14)	7144 (64.3)	129,764
71–80	1153 (10.38)	5991 (53.9)	108,821
81–90	1102 (9.92)	4889 (44.0)	88,804
91–100	939 (8.45)	3950 (35.5)	71,748
101–110	794 (7.14)	3156 (28.4)	57,326
111–120	628 (5.65)	2528 (22.7)	45,919
121–130	548 (4.93)	1980 (17.8)	35,965
131–140	411 (3.70)	1569 (14.1)	28,500
141–150	332 (2.99)	1237 (11.1)	22,469
151–160	277 (2.49)	960 (8.6)	17,438
161–170	203 (1.83)	757 (6.8)	13,750
171–180	192 (1.73)	565 (5.1)	10,263
181–190	141 (1.27)	424 (3.8)	7702
191–200	97 (0.87)	327 (2.9)	5940
Total	11,113	N/A	N/A

∗Percentage of patients with levels greater than or equal to the corresponding range × the total number of cirrhosis patients with HE in 2018, estimated as *N* = 201,858. HE: hepatic encephalopathy; N/A: not applicable.

**Table 5:** Proportion of HE patients with HE event.

Event count category	Number (%) of patients with HE (Symphony Health APLD claims database)	Number (%) of patients with HE (serum ammonia analysis cohort)
1 event	19,271 (32.6)	1683 (27.2)
2 events	8895 (15.0)	1194 (19.3)
3 events	6204 (10.5)	790 (12.8)
4 events	4575 (7.7)	550 (8.9)
5 or more events	20,225 (34.2)	1970 (31.8)

APLD: anonymized patient-level data; HE: hepatic encephalopathy.

## Data Availability

The data sets generated and analyzed for this manuscript are not publicly available. Requests for additional information should be directed to the study sponsor at medinfo@mnk.com. Individual de-identified patient data will not be disclosed.
